# Overlapping community finding with noisy pairwise constraints

**DOI:** 10.1007/s41109-020-00340-9

**Published:** 2020-12-11

**Authors:** Elham Alghamdi, Ellen Rushe, Brian Mac Namee, Derek Greene

**Affiliations:** University College Dublin, Dublin, Ireland

**Keywords:** Overlapping community finding, Community detection, Overlapping communities, Semi-supervised learning, Noisy pairwise constraints, Autoencoder (AE), Outlier detection, Deep learning

## Abstract

In many real applications of semi-supervised learning, the guidance provided by a human oracle might be “noisy” or inaccurate. Human annotators will often be imperfect, in the sense that they can make subjective decisions, they might only have partial knowledge of the task at hand, or they may simply complete a labeling task incorrectly due to the burden of annotation. Similarly, in the context of semi-supervised community finding in complex networks, information encoded as pairwise constraints may be unreliable or conflicting due to the human element in the annotation process. This study aims to address the challenge of handling noisy pairwise constraints in overlapping semi-supervised community detection, by framing the task as an outlier detection problem. We propose a general architecture which includes a process to “clean” or filter noisy constraints. Furthermore, we introduce multiple designs for the cleaning process which use different type of outlier detection models, including autoencoders. A comprehensive evaluation is conducted for each proposed methodology, which demonstrates the potential of the proposed architecture for reducing the impact of noisy supervision in the context of overlapping community detection.

## Introduction

Complex networks occur in many aspects of life, from social systems to biological processes. Despite their diversity, many networks share common properties and principles of organization (Boccaletti et al. 2006). One essential property that helps us to understand complex networks is the idea of *community structure*. Finding these sets of nodes or *communities* provides us with three important capabilities: understanding the structures and functionalities, modeling the dynamic processes in networks, and predicting their future behaviors. Generally, algorithms for detecting communities are unsupervised in nature. That is, they rely solely on the network topology during the detection process, rather than using any prior information or training data regarding the “correct” community structure. One common issue is that these algorithms can fail to uncover groupings that accurately reflect the ground truth in a specific domain, particularly when these communities highly overlap with one another (Ahn et al. 2010).

Recent work has improved the effectiveness of such algorithms by employing ideas from semi-supervised learning (Alghamdi and Greene 2019). This involves harnessing existing background knowledge (e.g. from domain experts or crowdsourcing platforms), which can provide limited supervision for community detection. Often this information takes the form of pairwise constraints between nodes (Basu et al. 2004a). Typically, pairwise constraints are either must-link and cannot-link pairs, indicating that either two nodes should be assigned to the same community or should be in different communities. As an example, we might be interested in finding social groups based on common interests on social media platforms, such as Facebook or Twitter, in order to target the most influential member of each social group for marketing and recommendation purposes. To improve our ability to achieve this, and go beyond simply looking at connections, we could use a human annotator, to query whether two users should be in the same group or different groups and label them as must-link or cannot-link, then incorporate these labels as constraints into community detection algorithms. By using this kind of knowledge, we can potentially uncover communities of nodes which are otherwise difficult to identify when analyzing complex networks.

Despite the promise of semi-supervised learning, in many real applications the supervision coming from human annotators will be unreliable or “noisy”. For instance, this might occur when using annotation acquired by crowdsourcing platforms (Howe 2008) such as Amazon Mechanical Turk (Kittur et al. 2008). In general human oracles will often be “imperfect”, in the sense that they can make subjective decisions, they may disagree with one another, they might only have limited knowledge of a domain, or they may simply complete a labeling task incorrectly due to the burden of annotation (Amini and Gallinari 2005; Du and Ling 2010; Sheng et al. 2008). Thus, when such judgements are encoded as pairwise constraints for semi-supervised community detection can be unreliable or conflicting, which can create problems when used to guide community finding algorithms (Zhu et al. 2015).

In this study, we explore the effect of noisy, incorrectly-labeled constraints on the performance of semi-supervised community finding algorithms for overlapping networks. To mitigate such cases, we treat the noisy constraints as outliers, and use an outlier detection strategy to identify and remove them, which has the effect of “cleaning” the constraints coming from the human oracle. The primary contributions of the paper are as follows:

1. We introduce a general architecture for semi-supervised community finding which incorporates a cleaning methodology to reduce the presence of noisy pairwise constraints, using outlier detection. This architecture can be implemented with any semi-supervised community finding that might involve querying an imperfect oracle. In this study, we focus the use of the AC-SLPA algorithm (Alghamdi and Greene 2018).
2. We propose alternative designs for cleaning methodology, based on different outlier detection models. Each design involves executing two parallel processes to separately reduce noise from must-link constraints and cannot-link constraints.
3. We investigate the performance of combining conventional outlier detection models and deep learning models for identifying noisy constraints.
4. We conduct comprehensive experiments to evaluate these alternative cleaning methods, as individual components, and when integrated within the proposed general architecture on a range of synthetic and real-world networks containing overlapping community structure.

The remainder of this paper is structured as follows. Section “Related work” provides a summary of relevant work in semi-supervised learning, in the context of both cluster analysis and community finding. In Section “Methods”, we describe the proposed general architecture for community detection which incorporates a cleaning process to reduce noise levels in pairwise constraints, and we propose multiple designs for implementing the cleaning process. In Section “Evaluation”, we discuss four experimental evaluations of these methods. Finally, we conclude our work in Section “Conclusion” with suggestions for further extending this work in new directions.

## Related work

To provide context for our work, this section describes related research of semi-supervised techniques in community finding, along with studies that address noisy pairwise constraints in both clustering and community finding.

### Semi-supervised learning in community finding

Several types of prior knowledge have been used in semi-supervised strategies to guide the community detection process. The most widely-used approach has been to employ pairwise constraints, either *must-link* or *cannot-link*, which indicate that either two nodes must be in the same community or must be in different communities. This strategy has been implemented via several algorithms, including modularity-based methods (Li et al. 2014), spectral partitioning methods (Habashi et al. 2016; Zhang 2013), a spin-glass model (Eaton and Mansbach 2012), matrix factorization methods (Shi et al. 2015), and various other methods (Yang et al. 2017; Zhang et al. 2019). Such approaches have often provided significantly better results on benchmark data, when compared to standard unsupervised algorithms.

Other authors have used different kinds of prior knowledge to provide supervision for community detection. For instance, Ciglan and Nørvåg (2010) developed an algorithm for finding communities with *size constraints*, where the upper limit size of communities is given as a user-specified input. This algorithm is based on standard label propagation methods for finding disjoint communities. In Wu et al. (2016) an optimization algorithm based on *density constraints* was proposed. This algorithm constructs an initial skeleton of the community structure by maximizing a criterion function that incorporates constraints to only find communities with intra-cluster densities above a given threshold. The remaining nodes are subsequently classified with respect to this skeleton. Other algorithms have used *node labels* as prior knowledge to improve the performance of community detection, using an approach which resembles traditional training data in classification (Leng et al. 2013; Liu et al. 2014; Wang et al. 2015). Liu et al. (2015) developed a method that uses a semi-supervised label propagation algorithm based on node labels and negative information, where a node is deemed not to belong to a specific community.

The majority of algorithms in this area have been designed to only find non-overlapping communities, where each node can only belong to a single community. However, many real-world networks naturally contain overlapping community structure (Adamcsek et al. 2006). To the best of our knowledge, little work has been done in the context of finding overlapping communities from a semi-supervised perspective. Dreier et al. (2014) performed some initial work here, using supervision for the purpose of algorithm initialization. Specifically, a small set of seed nodes was selected, whose affinities to a community was provided as prior knowledge in order to infer the rest of the nodes’ affinities in the network. On the other hand, Shang et al. (2017) used an expansion method that classifies edges into communities, where this model is trained on set of predefined seeds. However, there is no external human supervision used during the seed selection or expansion processes. In contrast, for our study, we focus on the problem of semi-supervised community detection based on the external supervision by human who are part of the networks or domain experts, and encode it as pairwise constraints since they have proven to be effective in a range of other learning contexts (Basu et al. 2004c; Greene and Cunningham 2007).

### Noisy constraints in clustering and community finding

Various algorithms have been proposed for the general task of pairwise constrained clustering, based on a variety of different clustering paradigms (e.g. Basu et al. 2004d; Davidson and Ravi 2005; Li et al. 2009). However, most assume the existence of “perfect” pairwise constraints which will be clean and will not contradict one another. Fewer studies have considered the requirement to handle noisy pairwise constraints. However, some relevant work in clustering has involved the development of new algorithms which are robust to noisy or conflicting pairwise constraints (Basu et al. 2004b; Coleman et al. 2008; Liu et al. 2007; Pelleg and Baras 2007). Other studies have introduced new metrics to assess the quality of constraints, considering aspects such as their informativeness and coherence (Davidson et al. 2006; Wagstaff et al. 2006). These can be used to filter or clean the pairwise constraints prior to clustering. A related study (Zhu et al. 2015) proposed an approach for handling noise by using a random forest classifier to identify incoherent constraints.

In contrast, in the field of semi-supervised community finding, the issue of noisy pairwise constraints has rarely been studied, and algorithms generally assume the veracity of any supervision supplied by an oracle. One related study from Li et al. (2014) initiated the work of handling “conflicting” pairwise constraints in non-overlapping community finding. That is, cases where ($v_{i}, v_{j}$) $\in$ *must-link*, ($v_{i}, v_{k}$) $\in$ *must-link*, and ($v_{j}, v_{k}$) $\in$ *cannot-link*. Such cases of conflict were identified using a dissimilarity index metric to measure the reliability of constraint pairs. However, this type of constraint conflict is in fact legitimate in the context of overlapping communities, as shown in our previous work in Alghamdi and Greene (2018). Therefore, the challenge remains of handling noisy constraints for overlapping community finding in an appropriate manner, which we seek to address in the next section.

## Methods

### Overview

Before describing our proposed architecture, we first provide a formal definition for the pairwise constraints which are used in this study. These definitions map to those which are widely adopted in the wider semi-supervised learning literature (Chapelle et al. 2006). Given a set of nodes *V* in a network, we define two constraint types:

1. A *must-link constraint* specifies that two nodes should be assigned to the same community. Let *ML* be the must-link constraint set: $\forall$ $v_{i}, v_{j}$ $\in$ *V* where *i* $\ne$ *j*, then the constraint ($v_{i}, v_{j}$) $\in$ *ML* indicates that two nodes $v_{i}$ and $v_{j}$ must be assigned to the same community.
2. A *cannot-link constraint* specifies that two nodes should **not** be assigned to the same community. Let *CL* be the cannot-link constraint set: $\forall$ $v_{i}, v_{j}$ $\in$ *V* where *i* $\ne$ *j*, then the constraint ($v_{i}, v_{j}$) $\in$ *CL* indicates that $v_{i}$ and $v_{j}$ must be assigned to two different communities.

As discussed in Alghamdi and Greene (2019), implementing pairwise constraints in the context of overlapping communities is challenging due to the lack of the *transitive property* for must-link constraints in the context of overlapping communities. In the case of non-overlapping communities, must-link constraints have a transitive property, where a third must-link relationship can be inferred from two other associated must-link constraint pairs. For instance, if ($v_{i}, v_{j}$) $\in$ *ML*, and ($v_{i}, v_{k}$) $\in$ *ML*, then we can also infer that ($v_{j}, v_{k}$) $\in$ *CL*. This property does not hold for overlapping communities. For instance, node $v_{i}$ might be an overlapping node and in this case there are two possible scenarios for the pair ($v_{j}, v_{k}$): (1) ($v_{j}, v_{k}$) $\in$ *CL* where node $v_{i}$ might be an overlapping node that have a must-link constraint with both $v_{j}$ and $v_{k}$, yet these two nodes could belong to two different communities; (2) ($v_{j}, v_{k}$) $\in$ *ML* where all three nodes are in fact in the same community. This problem has been addressed in detail in Alghamdi and Greene (2019) and therefore is not the main focus of this paper.

**Fig. 1:**
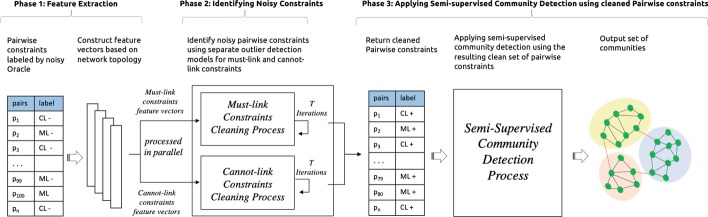
An illustration of the overall pairwise constraint cleaning process

Now we describe our proposed general architecture for semi-supervised community detection which incorporates a methodology to reduce the presence of noisy pairwise constraints using an outlier detection model, as illustrated in Fig. 1. This architecture begins with a set of noisy pairwise constraints provided by a human oracle ($PC-$). The set of noisy pairwise constraints ($PC-$) is composed of must-link ($ML-$) and cannot-link ($CL-$) constraints. These constraints are cleaned to produce a revised set of constraints ($PC+$) (composed of must-link ($ML+$) and cannot-link ($CL+$) constraints) which are fed into the community finding process. The proposed architecture consists of three distinct phases:

1. **Phase 1: Feature extraction.** After receiving a set of pairwise constraints ($PC-$) from a potentially-noisy oracle, features vectors are constructed to provide inputs to outlier detection models later, with one vector per constraint pair (for both must-link and cannot-link). These vectors encode various aspects of the relationship between a pair of nodes according to the underlying network topology. These features include standard measures based directly on the network, including: whether the pair of nodes share an edge, their number of common neighbors, the shortest path length between them, and their cosine similarity. We also include more complex features, such as their *SimRank* similarity (Jeh and Widom 2002), and their similarity as computed on a *node2vec* embedding generated on the network (Grover and Leskovec 2016).
2. **Phase 2: Identifying noisy constraints.** This involves executing two parallel processes that use two different outlier detection models to separately eliminate noise from the original must-link set ($ML-$) and cannot-link set ($CL-$). The constructed feature vectors are fed into each model for multiple iterations of cleaning, returning a score for each constraint that determines whether or not it is an outlier (i.e. a noisy constraint).
3. **Phase 3: Applying Semi-supervised Community Detection Process.** The returned clean pairwise constraint set ($PC+: ML+,CL+$) is passed to a semi-supervised community detection algorithm to be used during the process of finding communities.

In the following sections, we describe the details of the proposed architecture in terms of the outlier detection methods used to identify potentially-noisy constraints (Section “Outlier detection methods”), the different variations of the second phase of the architecture shown in Fig. 1 (see Section “Process for identifying noisy constraints”), and the implementation of the proposed architecture in the context of the AC-SLPA community finding algorithm (see Section “AC-SLPA with noise identification”).

### Outlier detection methods

**Isolation Forests:** This method, proposed by Liu et al. (2008), uses a tree-based ensemble strategy for anomaly detection. The assumption underlying this method is that anomalies will be isolated earlier in their trees as these examples are not only rare, but also have feature values substantially different from the normal data. Random partitions are used in order to separate examples, with the number of partitions acting as the path length. Because anomalous feature values are assumed to significantly differ from that of normal examples, these features will more easily split anomalous examples from normal examples early on in the tree, leading to a shorter path. This shortening effect is compounded by the fact that these examples are also assumed to be rare. In order to compute an anomaly score, the average path length over multiple trees is computed, and normalized by the average path length over all paths. Scores close to 1 are said to be anomalous and scores close to 0 are assumed normal. This algorithm fits the problem of noise detection when there are far fewer noisy labels than normal examples.

**One-class SVM:** One class Support Vector Machine (OCSVM) (Schölkopf et al. 2000, 2001) is a commonly-used method for anomaly detection which extends support vector algorithms to one-class classification. The reference to “one class” here refers to the assumption that primarily data from the normal class (i.e. non-outliers) will be modeled during training. First, data is transformed by a map $\phi$ to a higher dimensional space by evaluating a kernel function. The algorithm then seeks to find the separating hyperplane in the kernel space between data and the origin with the largest margin. This is achieved by solving the following quadratic program for given training examples ${\pmb {x}}_1,{\pmb {x}}_2,\ldots ,{\pmb {x}}_l$:

1 $$\begin{aligned} \min \frac{1}{2} \left\| w \right\| ^2 + \frac{1}{\nu l} \sum ^l_i \xi _i - p \end{aligned}$$

subject to

2 $$\begin{aligned} (w \cdot \phi ({\pmb {x}}_i)) \geqslant p - \xi _i, \xi _i \geqslant 0 \end{aligned}$$

where *w* and *p* solve the problem. Here, $\xi _i$ refers to the slack variable for a given example ${\pmb {x}}_i$ which softens the margin, allowing for some points to reside outside the margin, essentially relaxing the assumption of complete separability between normal and outlying data. The hyperparameter $\nu \in (0,1)$ controls the number of outliers with smaller values allowing outliers to have a greater affect on the decision function. The decision function is given by

3 $$\begin{aligned} f({\pmb {x}})= sgn((w \cdot \phi ({\pmb {x}})) - p) \end{aligned}$$

where *sgn*(*z*) outputs a value of $+1$ for $z \geqslant 0$, indicating normal data and $-1$ otherwise, indicating an outlier.

**Local Outlier Factor:** This method is based on the concept of *local density* in detecting outliers. Given a particular point *p*, we measure the density of *p* with respect to the density of its *k* nearest neighbors. Intuitively, if the local density of *p* is lower than the local densities of its neighbors, this indicates that *p* is an outlier. As discussed in Breunig et al. (2000), for a given neighborhood size *k*, the *k-distance(p)* for a point *p* is defined as the distance between *p* and its *k*-th neighbor *o* (i.e. the *k*-th closest point to *p*). The *k-distance neighborhood* $N_k(p)$ is the set of points whose distances do not exceed the *k-distance(p)*. The *reachability distance* is then defined as:

4 $$\begin{aligned} reachdist_k(p,o) = max\{k-distance(o), d(p,o)\} \end{aligned}$$

This means that if *p* is *o*’s *k*-th nearest neighbor, this will be returned, otherwise, the true distance between *p* and *o* will be returned. In order to calculate the densities of different clusters of points, the “local reachability density” $lrd_k$ is calculated.

5 $$\begin{aligned} lrd_k(p) = 1/ \frac{\sum ^k_{o_i} reachdist_k(p,o) }{ |N_k(p)|} \end{aligned}$$

Finally, the local outlier factor (LOF) of point *p* is defined as:

6 $$\begin{aligned} LOF_k(p) = \frac{\sum ^k_{o_i} (\frac{lrd_k(o)}{lrd_k(p)}) }{|N_k(p)|} \end{aligned}$$

**Autoencoders:** An autoencoder (AE) represents a type of neural network architecture that attempts to reconstruct a given input in an effort to learn an informative latent feature representation. Formally, for an input vector *x*, an attempt is made to find a mapping from *x* to a reconstruction of itself $x^\prime$ . By doing this, a latent representation of the data is created in the hidden layer(s) of the network. The general form of a single hidden layer autoencoder as follows:

7 $$\begin{aligned} f(x) =\sigma (x,W^e), \quad g(z) =\sigma (z,W^d), \quad \text {and}\quad x ^\prime =g(f(x)) \end{aligned}$$

where *f*(*x*) is the encoder function for input *x*, *g*(*z*) is decoder function for encoding *z*, $\sigma$ is a non-linear function, $W^e$ and $W^d$ are weight matrices for the encoder and decoder respectively and $x ^\prime$ is the reconstruction of the input vector (Goodfellow et al. 2016).

These networks can use a “bottleneck” configuration where the hidden layer(s) of the network compress the data (Goodfellow et al. 2016). The network is trained by minimizing the mean squared error (MSE) between the reconstruction and input. as shown in formal (8):

8 $$\begin{aligned} MSE(x,x ^\prime ) = \frac{1}{n}\sum _{i=1}^{n}{(x_{i}-x_{i}^\prime )^2} \end{aligned}$$

Additionally, autoencoders can be constrained to enforce sparsity in the network and therefore no longer require a compressed network capacity. One type of constrained autoencoder adds a sparsity penalty to hidden representations by constraining their absolute value. This penalty term is weighted and added to the cost function. The constrained cost is defined as.

9 $$\begin{aligned} MSE(x,x ^\prime ) = \frac{1}{n}\sum _{i=1}^{n}{(x_{i}-x_{i}^\prime )^2 + \lambda \sum _{i}|h_i|} \end{aligned}$$

where $\lambda$ is the sparsity penalty and $h = f(x)$ (Goodfellow et al. 2016).

Autoencoders can be used in a number of capacities. In this work, we propose a number of techniques for noise detection from pairwise constraint sets which make use of autoencoders in different ways. Firstly, we show that autoencoders can be used as an effective outlier detection technique for noise detection in pairwise constraints. Secondly, we demonstrate that autoencoders can also be used as an embedding method to support other outlier detection methods in the identification of noisy constraints.

### Process for identifying noisy constraints

**Fig. 2:**
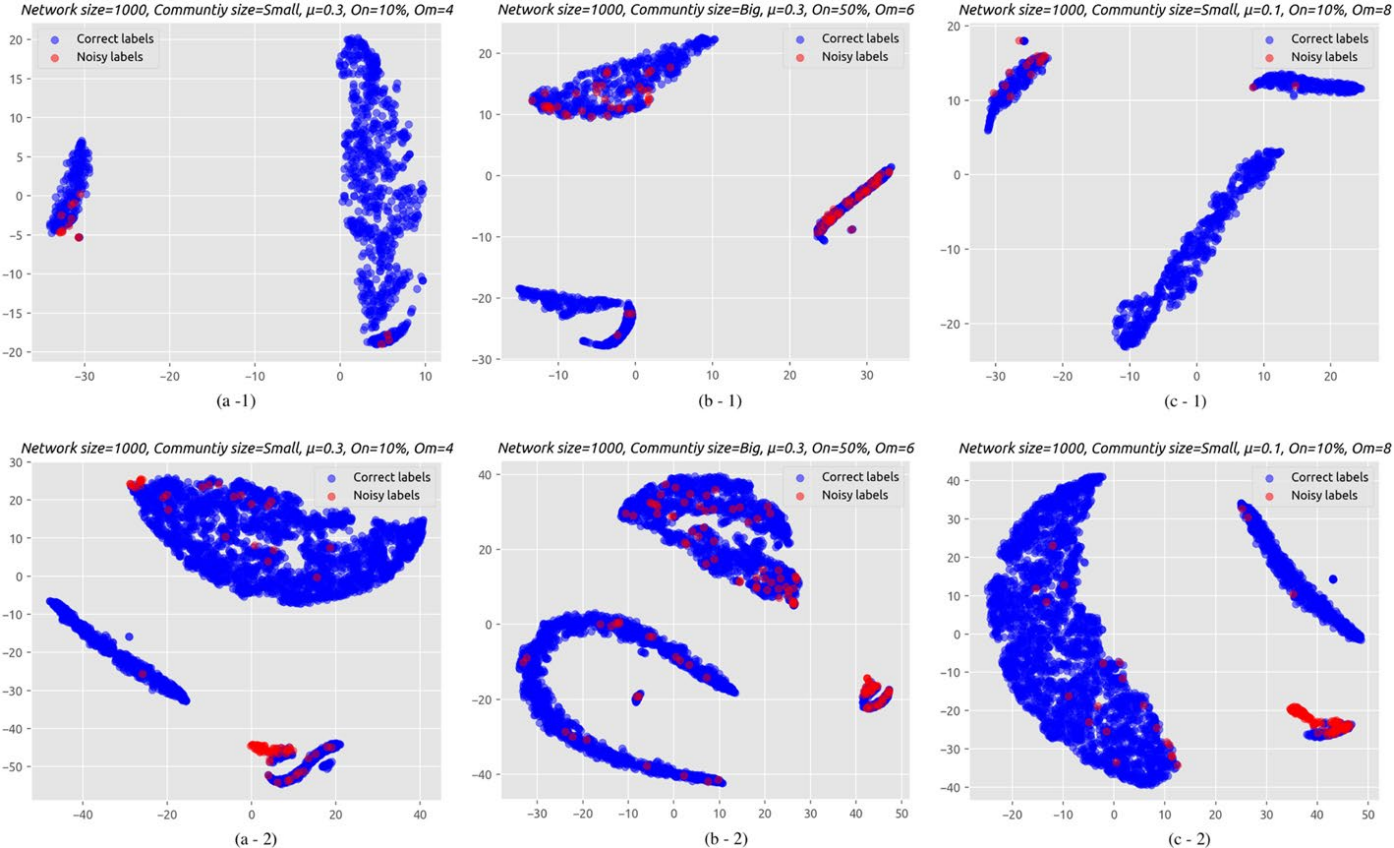
An illustration of the differences between the distributions of must-link and cannot-link constraints, as viewed in a low-dimensional space, for a sample of small networks. The plots in the first row (a-1, b-1, c-1) show the must-link constraints of a sample set of small synthetic networks. The second row (a-2, b-2, c-2) shows the cannot-link constraints of the same set of networks

In this section, we describe a number of alternative cleaning processes for reducing noise in pairwise constraints, before passing them to a semi-supervised community detection algorithm. These cleaning processes employ some of the outlier detection models described in Section “Outlier detection methods”. It is important to note that pairwise constraints are of two distinct types: must-link and cannot-link. The differences in their respective distributions, which can be seen in Fig. 2, motivates the use of two separate cleaning processes and exploring different outlier detection models for each. The selection of models is based on best performance in detecting noises in constraints as illustrated in the evaluation section.

**Fig. 3:**
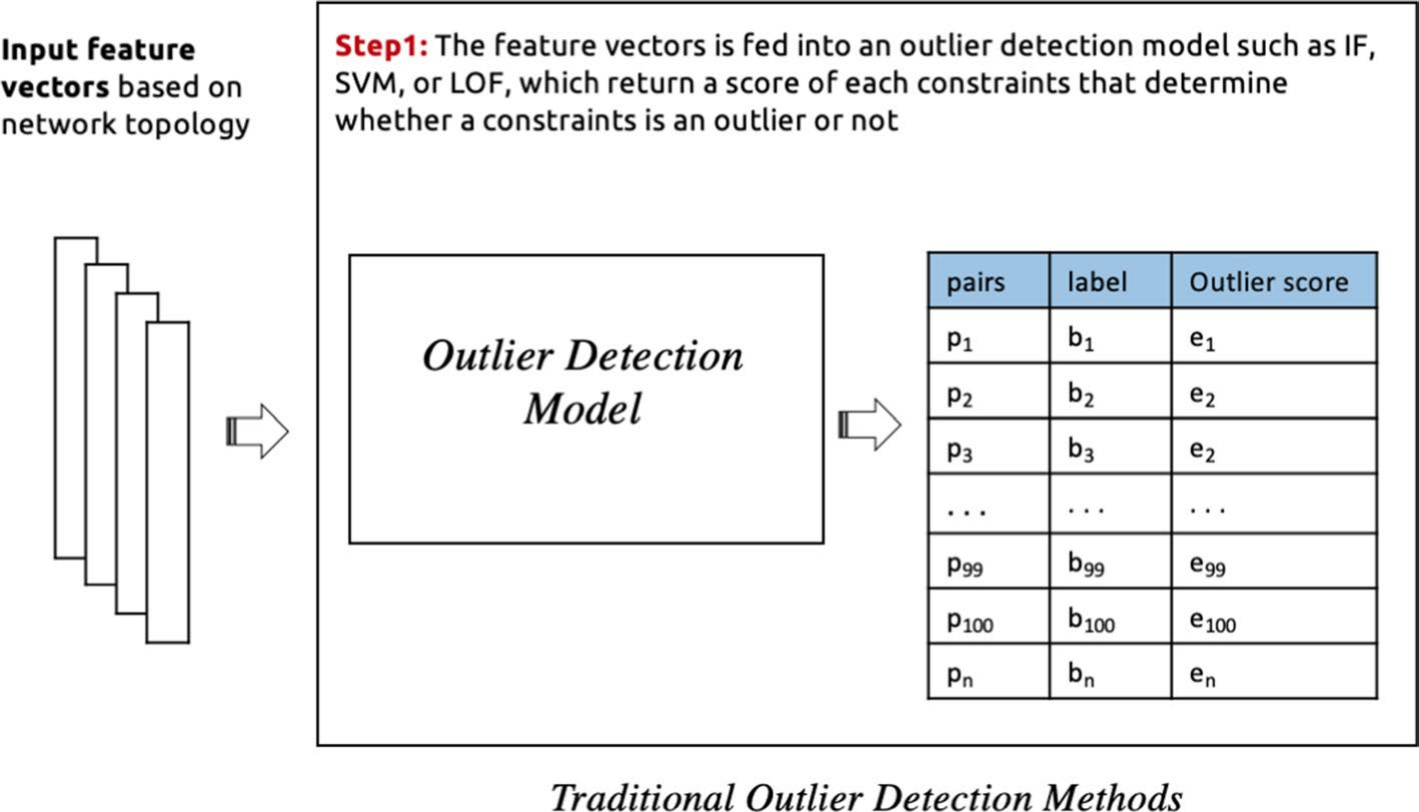
An illustration of the process for identifying noisy pairwise constraints using traditional outlier detection

**Fig. 4:**
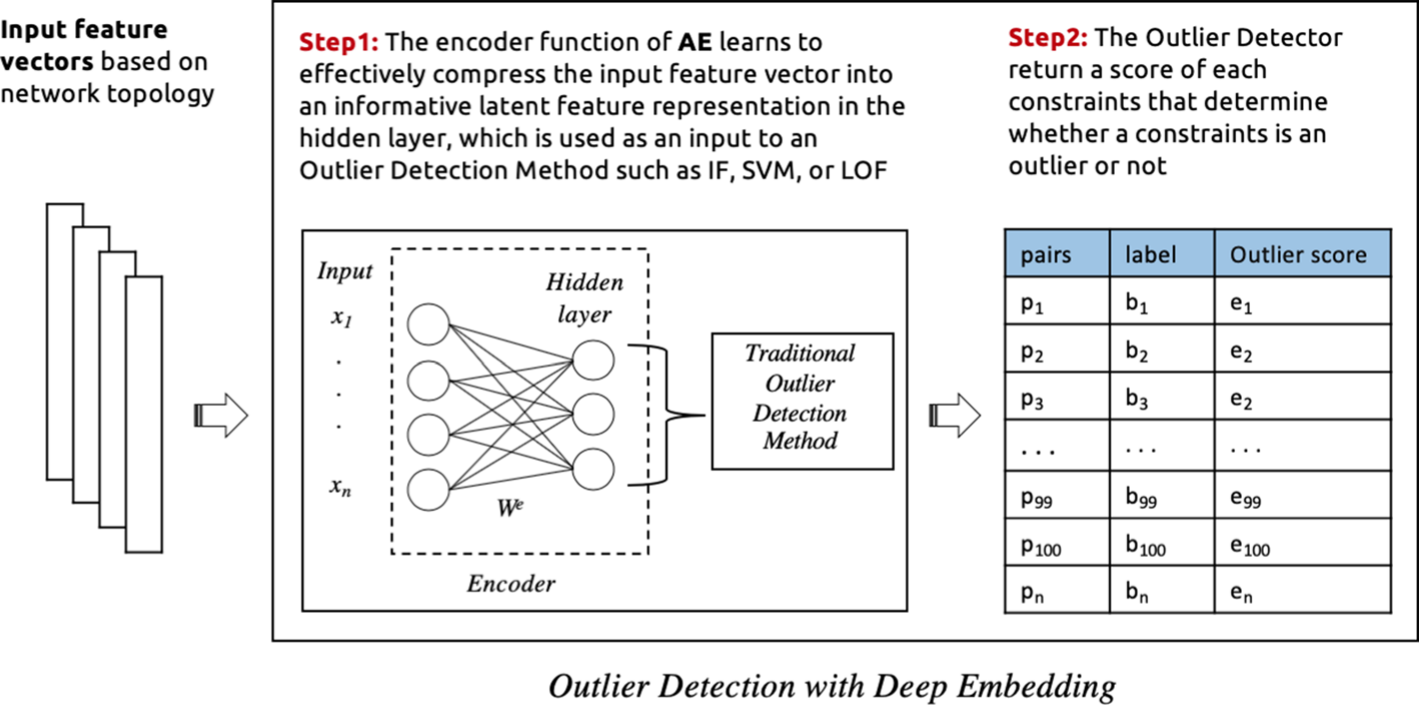
An illustration of the process for identifying noisy pairwise constraints using outlier detection via deep embedding function

**Fig. 5:**
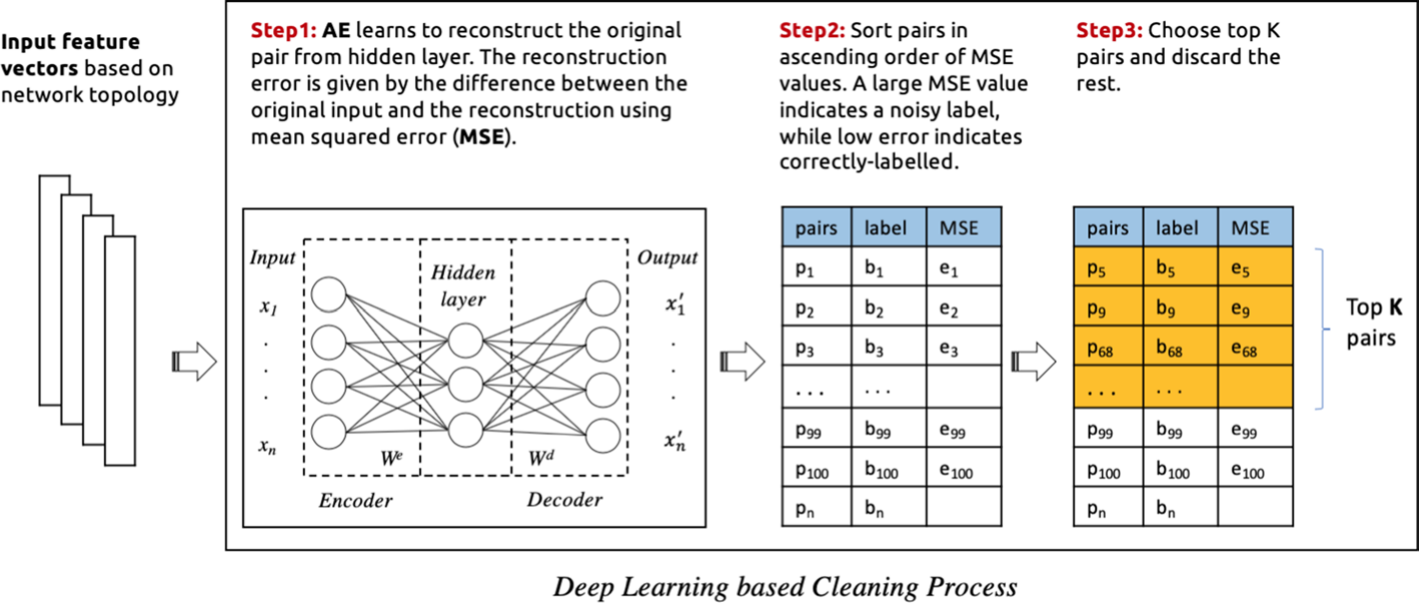
An illustration of the process for identifying noisy pairwise constraints using deep learning approach (neural network autoencoder (AE))

**Fig. 6:**
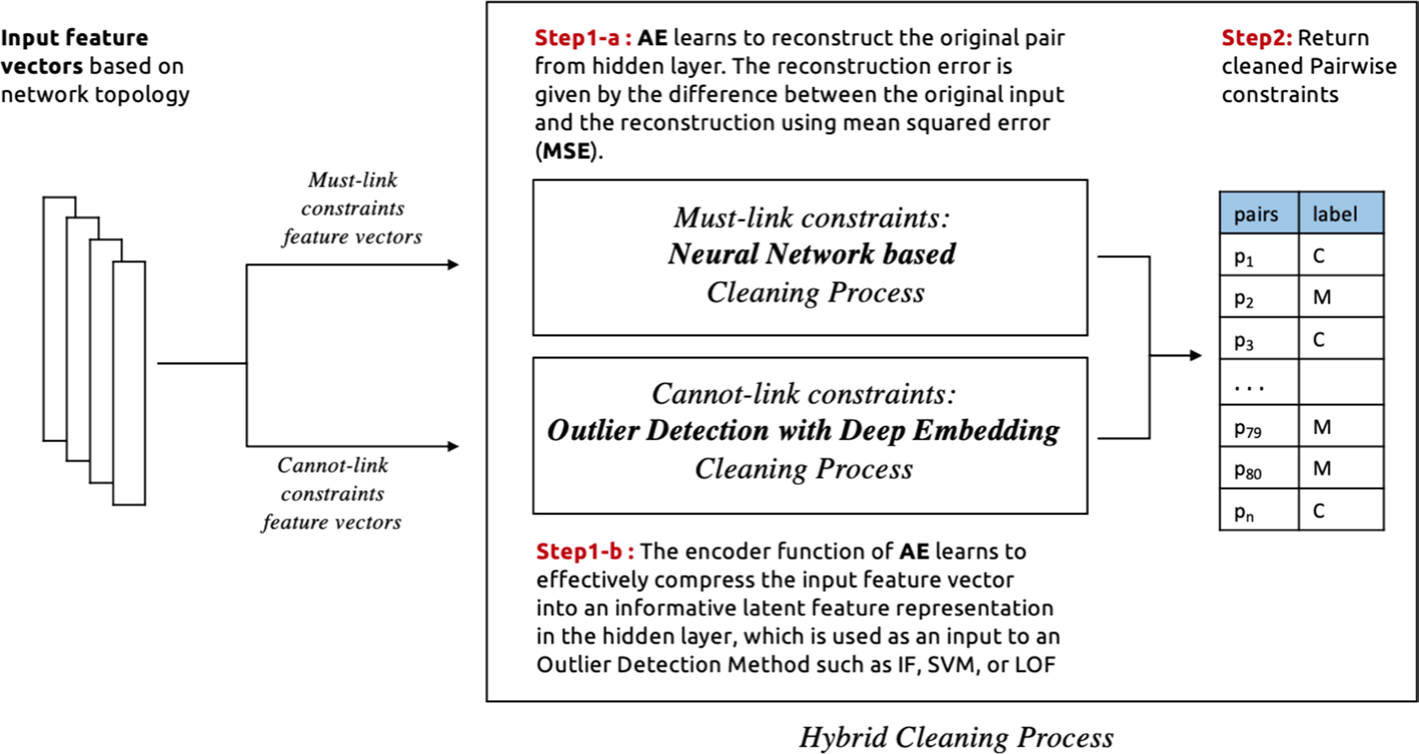
An illustration of the process for identifying noisy pairwise constraints using hybrid cleaning process. A combination of traditional models, and deep learning based outlier detection models

In this study, we explore the implementation of the following cleaning processes which are classified into four categories based on the employed outlier detection model:

1. **Traditional outlier detection:** In this process, a stand-alone outlier detection method is selected (e.g. isolation forest, One-class SVM, local outlier factor) to identify noise in must-link and cannot-link sets separately. The input features are passed to these models, which then return a binary score for each constraint which determines whether or not it is a noisy constraint. See Fig. 3 for an illustration.
2. **Outlier detection via deep embedding:** Here the neural network autoencoder (AE) is used as an additional component to provide an embedding function for a traditional outlier detection method. In this case, only the encoder function from the autoencoder model is used. After feeding the feature vectors into the encoder function, the model learns to effectively compress the input feature vector into an informative latent feature representation in the hidden layer. Then this latent representation is used as an input to an outlier detection method such as Isolation forest, One-class SVM, or local outlier factor, which return a binary score that identify the noisy constraints. See Fig. 4 for an illustration. This process is conducted for must-link and cannot-link pairs separately with different encoder functions and outlier detection methods. The selection of models is based on experimental results as illustrated in the evaluation section.
3. **Deep learning approach:** In this case, the neural network autoencoder (AE) is used as an outlier detection technique for identify noises in pairwise constraints. Different autoencoder models is used for must-link and cannot-link pairs separately. The feature vectors are fed into the autoencoder model, which learns to reconstruct the original constraints from the latent representation. The reconstruction error is given by the difference between the original constraints and the reconstruction. A large error is indicative of an outlier (i.e. a noisy constraint), while a low error indicates a “normal” example (i.e. a correctly-labelled constraint). Finally, we sort the constraints in ascending order (lowest to highest error) in order to determine the top *k* constraints with the lowest level of error. The expectation is that, as the larger part of pairwise constraints are non-noisy, the autoencoder’s latent representation will be biased towards these examples. This makes the model somewhat robust to outliers. Based on this property, it is then assumed that examples which are noisy will have a high reconstruction error. See Fig. 5 for an illustration of the process.
4. **Hybrid cleaning process:** For each of the above described cleaning processes, we use separate processes of the same category to identify noises in must-link and cannot-link pairs. However, in this process, we investigate a combination of different categories processes for must-link and cannot-link pairs. Based on initial experiments, a Neural Network based cleaning process performed better for must-link pairs than cannot-link. On the other hand, using Outlier Detection with Deep Embedding for cannot-link pairs is found to yield better noise detection performance, when compared to using an autoencoder alone. See Fig. 6 for an illustration of the process.

We see from Fig. 2 that the distributions of correct labels and noisy constraints is more complex in the case of cannot-link constraints—i.e., there is a high overlap between both the correct and noisy groups. Separating these groups requires a more complex function, as compared to the equivalent case for must-link constraints, which are relatively easy to separate.

### AC-SLPA with noise identification

Now we discuss the implementation of the general architecture discussed in Section “Overview” in the context of the existing AC-SLPA algorithm (Alghamdi and Greene 2018) in order to create a robust active semi-supervised SLPA algorithm that can handle the presence of noisy pairwise constraints. The new modified AC-SLPA consists of three stages. The first two stages include the pairwise constraints cleaning process, which are executed iteratively as follows:

**Stage 1: Detecting noises in constraints during selection and annotation.** At each iteration of AC-SLPA, informative pair of nodes are selected using *Node Pair Selection* method (Alghamdi and Greene 2019) and passed to the noisy oracle to be labelled as pairwise constraints. After generating a set of noisy pairwise constraints ($PC-$), this set is passed to the process of identifying noisy constraints for multiple sub-iterations of cleaning. As a new set of constraints is introduced at each iteration, the outlier detectors are retrained at each one of these iterations and reapplied to the remaining set of constraints. The output constraints of this process are then used to apply PC-SLPA algorithm. At the end of each run of AC-SLPA, the cleaned pairwise constraint set ($PC+$) is accumulated and mixed with the new chunk of noisy pairwise constraints ($PC-$) in the next iteration. The larger the constraints set passed to the outlier detection model, the better the performance.

**Stage 2. Rechecking discarded pairwise constraints.** The previous stage of cleaning may result in a number of non-noisy constraints being labelled as noisy. This is more likely to happen when the distribution of noisy constraints is highly overlapped with non-noisy constraints. The second stage is designed to recheck the discarded pairwise constraints set ($PC-$) that were potentially mislabelled as noises, by passing them to the process of identifying noisy constraints for another multiple iterations of cleaning, thus reducing any wastage of the annotation budget. The returned set of constraints from this process is added to the accumulated cleaned pairwise constraints set ($PC+$) from stage 1.

**Stage 3. Apply PC-SLPA.** The final stage involves applying the semi-supervised community detection process PC-SLPA using the final accumulated cleaned pairwise constraints ($PC+$) obtained from the previous two stages, thus producing a final set of communities. The complete architecture is summarized in Algorithm 1. 
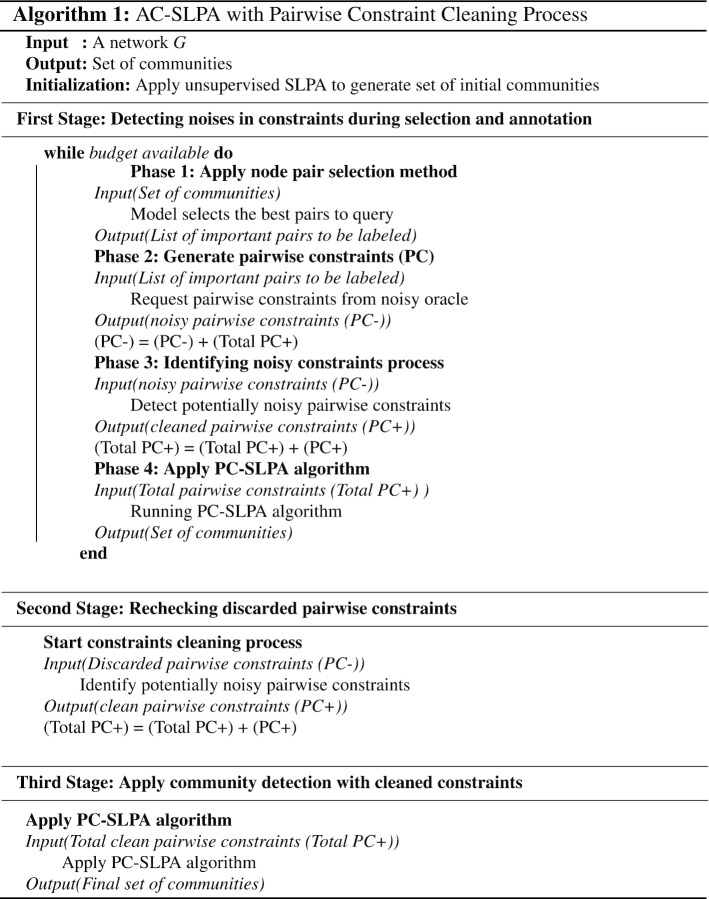


## Evaluation

In this section, we describe the datasets and experimental configuration used to validate our proposed method for handling noisy constraints. We conduct four experiments to show its effectiveness, which are applied to synthetic benchmark networks of different sizes with overlapping communities, and real-world networks. Our objectives are as follows: (1) to quantify the ability of each constraint cleaning process to detect noisy constraints prior to community finding; (2) to choose the best architectures of autoencoder to use as a deep embedding function for outlier detection models; (3) to compare all types of constraint cleaning processes after integration with AC-SLPA, in order to evaluate the end-to-end performance of the complete architecture; (4) to examine the performance of the method on real-world data.

### Datasets

**Synthetic data.** We constructed a diverse set of 64 benchmark synthetic networks using the widely-used LFR generator (Lancichinetti et al. 2008). These networks vary in terms of number of nodes $N \in [1000, 5000]$, communities per node (overlapping diversity) $O_m \in [2,8]$, and the fraction of nodes belonging to multiple communities (overlapping density) $On \in \{10\%,50\% \}$. These networks contain either small communities ($10-50$ nodes), or large communities ($20-100$ nodes). The mixing parameter $\mu$ varies from 0.1 to 0.3, which controls the level of community overlap. Details of the network generation parameters are in Table 1.

**Table 1:** Parameter ranges used for the generation of LFR synthetic networks

Parameter	Description	Value	Parameter	Description	Value
*N*	Number of nodes	1000–5000	$t_1$	Degree exponent	2
*k*	Average degree	10	$t_2$	Community exponent	1
$K_{max}$	Max degree	50	$\mu$	Mixing parameter	0.1–0.3
$C_{min}$	Min community size	10/20	$O_n$	Num. overlapping nodes	10%/50%
$C_{max}$	Max community size	50/100	$O_m$	Communities per node	1–8

**Real-world data.** We use three real-world networks which contain annotated ground truth overlapping communities. These are: (1) a co-purchasing network from Amazon.com; (2) a friendship network from YouTube; (3) a scientific collaboration network from DBLP. These networks have previously been used in the community finding literature (Leskovec and Krevl 2015). For each network, we include only the 5000 largest communities, as performed in Yang and Leskovec (2015). We then conduct a filtering process as per Harenberg et al. (2014). The remaining communities are ranked based on their internal densities and the bottom quartile is discarded, along with any duplicate communities. As an additional step, we remove extremely small communities. For the Amazon and YouTube networks, communities of size $<5$ nodes are discarded, while for the DBLP network communities with $<10$ nodes are discarded. Details of the final networks are summarized in Table 2.

**Table 2:** Details of real-world networks

Real-world Networks	Amazon	YouTube	DBLP
#Nodes-# Edges-#Communities	7411-21214-876	6426-23226-1058	7233-33045-613
Average degree	5	7	9
Maximum community size	27	31	38
Minimum community size	5	5	10
Average community size	10	7	12
Maximum communities per node §($O_m$)	4	11	5
Number of overlapping nodes ($O_n$)	1394 (18%)	865 (13%)	214 (3.3%)
Clustering coefficient	0.74	0.33	0.90

**Constraint noise.** In all of our experiments we mimic the presence of an oracle by using pairwise node co-assignment information in the ground truth communities for each network. We use this information to create pairwise constraints, according to the definition of constraints given in Section “Overview”. We subsequently add noise to these constraints by flipping the labels of a randomly-selected subset of must-link and cannot-link pairs. The level of noise is fixed at $10\%$ of the smallest constraint set, either must-link or cannot-link.

**Evaluation metrics.** To compare the ability of autoencoders variants to detect noisy constraints before their use in community finding, we calculate the AUC (Area Under the ROC Curve) over the reconstruction error. This provides an estimate of the number of constraints that were successfully detected in the absence of a threshold. After integrating this step into the community finding process, performance is assessed using the overlapping form of Normalized Mutual Information (NMI) (Lancichinetti et al. 2009), which has been widely adopted in the literature (Xie et al. 2013). For this measure, a value close to 1 indicates a high level of agreement with the ground truth communities, while a value close to 0 indicates that the communities generated by an algorithm are no better than random.

Several alternative validation metrics have been proposed in the literature to capture the topological properties of a network. These are used to assess the quality of a set of communities when no ground truth communities are available, and include metrics such as modularity (Newman 2004) and its overlapping counterpart (Lázár et al. 2010).

Some studies have suggested measuring the topological features of communities generated by an algorithm, and then comparing the outputs to the ground truth communities in the network (Dao et al. 2020; Orman et al. 2012). This can be seen as a complementary evaluation to the more widely-adopted external metrics. These approaches involve considering factors such as community size distributions, average distance between all pairs of nodes within a community, and scaled community density. Later in Section “Experiment 4: Real-world networks”, we consider the analysis of community size distributions to provide an additional evaluation perspective on our proposed approach.

### Experiment 1: Comparing Outlier Detection Models

In this experiment, the objective is to find the best models for detecting noisy constraints in must-link and cannot-link sets in Phase 2 (identifying noisy constraints) of the proposed general architecture in Fig. 1. As described in Section “Process for identifying noisy constraints”, there are four categories of cleaning processes that can be used in Phase 2. This experiment is designed to find the best model for each category. There are three main aspects of this experiment:

1. In Section “Evaluating outlier detection methods” we seek to find the best autoencoder architectures as outlier detection models for must-link and cannot-link constraints separately. These will be used to investigate the deep learning approach as a cleaning processes in Experiment 2, Section “Experiment 2: Evaluation of noise removal methods”
2. Also in Section “Evaluating outlier detection methods” we identify the best performing conventional outlier detection method (from Isolation forest, One-class SVM, and local outlier factor) for must-link and cannot-link constraints. This outcome will also be used in Experiment 2, Section “Experiment 2: Evaluation of noise removal methods”.
3. In Section “Evaluating autoencoders for deep embeddings” we explore different autoencoder architectures as deep embedding technique integrated with conventional outlier detection models for each must-link and cannot-link constraints.

This experiment is designed to assess the performance of Phase 2 detached from the general architecture in Fig. 1. Specifically, constraints are selected over 10 independent iterations of the existing AC-SLPA algorithm and then split into must-link and cannot-link sets to be processed separately.

#### Evaluating outlier detection methods

**Methodology.** This experiment compares two different strategies for cleaning constraint sets, evaluated on the synthetic LFR networks described previously in Section “Datasets”. This experiment proceeds in the following steps:

1. We consider autoencoder models for constraint cleaning. For each selected set at each iteration, a separate autoencoder is trained on this set until the reconstruction error reaches a near-zero value (functionally a maximum number of epochs is selected). The set is then passed through the autoencoder once again in order to obtain a reconstruction for each constraint. The reconstruction error is then calculated for each constraint. The number of layers in each autoencoder model is also varied in order to examine whether this task benefits from a deeper model. Both compression-based autoencoders and sparse autoencoders are considered for this. In the case of the compression autoencoders, the nodes in the encoder are gradually decreased until the bottleneck layer is reached and then gradually increased in the decoder. For the L1 constrained autoencoders, compression in the encoder is not necessary, and therefore the dimensionality is kept the same as the input throughout the network. In the case of the constrained autoencoders, the sparsity weight is kept at $10^{-3}$. All models were trained with a batch size of 256. The full list of parameter combinations used in our experiments is given in Table 3. In the remainder of this paper we denote these autoencoder architectures with the prefix AE*.
2. As baseline alternatives, we consider traditional outlier detection methods for this task: Isolation Forest (IF) (Liu et al. 2008), One-class SVM (Schölkopf et al. 2000), and local outlier factor (Breunig et al. 2000). We conduct experiments in the same way as for the autoencoders described above. For each selected set at each iteration, a separate model is fit on this set, which then returns a binary score for each constraint that determines whether or not it is a noisy constraint. After removing noisy constraints, the same set is then passed through the model once again in order to obtain a re-calculated score for each constraint. We use the code released by Pedregosa et al. (2011), with the default parameter settings, including the contamination parameter at $10\%$.

**Table 3:** Details of autoencoder architectures. Here AE* indicates the number of layers in compression autoencoders, and AE*_L1 indicates the number of layers in L1-constrained autoencoders

Architecture	Nodes per layer	Small networks		Large networks	
		Epochs	Learning rate	Epochs	Learning rate
AE1	dim:(7,3,7)	100	0.01	30	0.001
AE1_L1	dim:(7,7,7)	100	0.01	30	0.001
AE2	dim:(7,5,3,5,7)	100	0.01	30	0.001
AE2_L1	dim:(7,7,7,7,7)	100	0.01	30	0.001
AE3	dim:(7,6,5,3,5,6,7)	100	0.01	30	0.001
AE3_L1	dim:(7,7,7,7,7,7,7)	100	0.01	30	0.001

**Results.** Tables 4 and 6 present the results for the two alternative strategies (autoencoders and standard outlier detection methods). Each table is divided into two parts that represent the average AUC scores of each model on small and large networks respectively. Results for must-link and cannot-link constraint sets are listed separately. Each table entry shows the average AUC score of the model (on the rows) for networks with certain size, overlapping density and the type of constraints used (on the columns). The highest average AUC score is highlighted in bold.

In terms of the autoencoder models, for both small and large network the most constrained AE models tend to perform better than the unconstrained ones when detecting noises on must-link constraints, as illustrated in Table 4. For instance on small networks, the average AUC score of AE2 is 0.625 and increases to 0.657 with the constrained version AE2_l1. Similarly, on large networks, AE2_l1 show a higher average score than AE2, with AUC = 0.470 and AUC = 0.442 respectively. In contrast, we see the opposite trend for cannot-link constraints, where constrained models show lower average scores than unconstrained ones, except for AE3_l1 which presents consistently higher score compared to AE3 in all cases.

**Table 4:** Average AUC scores for different autoencoder architectures. We report overall scores, and scores on networks with differing overlap density $O_n$, for must-link and cannot-link sets separately

Architecture	Small Networks						Large Networks					
	Must-link constraints			Cannot-link constraints			Must-link constraints			Cannot-link constraints		
	Overall	$O_n=10$%	$O_n=50$%	Overall	$O_n=10$%	$O_n=50$%	Overall	On=10%	$O_n=50$%	Overall	$O_n=10$%	$O_n=50$%
AE1	0.634	0.651	**0**.**616**	0.779	0.820	0.739	0.456	**0**.**452**	0.460	0.844	0.906	0.782
AE2	0.625	0.652	0.597	0.785	0.828	0.742	0.442	0.443	0.442	0.849	0.906	0.791
AE3	0.624	0.667	0.580	0.796	0.843	0.748	0.422	0.416	0.429	0.849	0.905	0.794
AE1_l	0.659	0.705	0.614	0.737	0.777	0.697	0.453	0.445	0.461	0.789	0.842	0.735
AE2_l	**0**.**657**	0.718	0.597	0.776	0.812	0.739	**0**.**470**	0.444	**0**.**496**	0.780	0.847	0.714
AE3_l	0.654	**0**.**773**	0.534	**0**.**835**	**0**.**877**	**0**.**793**	0.424	0.407	0.440	**0**.**879**	**0**.**919**	**0**.**839**
All AE	0.642	0.694	0.590	0.785	0.826	0.743	0.444	0.434	0.45	0.832	0.887	0.775

When comparing shallow to deep models on small and large networks, the general trend of AUC scores on must-link constraints shows a decrease as more layers are added to AE models, except for AE2_l1 on large networks. On the other hand, we can see a contrasting trend on cannot-link constraints, where the highest AUC scores on all networks are achieved by the deep model AE3_l1.

Interestingly, for both types of constraint, the AE models tend to perform significantly better on networks with low overlapping density. For instance, the average AUC scores for AE models is 0.694 for must-link constraints and 0.826 for cannot-link constraints on small networks with $O_n=10\%$, which are higher than AUC = 0.590 and AUC = 0.743 on $O_n=50\%$ for must-link and cannot-link constraints respectively. However, this excludes the results of AE models on must-link constraints for large networks, which show slightly higher scores.

Table 5 summarizes the average ranks of all AE models on must-link and cannot-link constraints separately for small networks and large networks. Each table entry shows the average rank (lower values are better) of a model (on the rows) over each constraint type and networks size (on the columns). The ranking scores indicate that, for must-link constraints, the best approaches for detecting noise are the shallow model AE1_l1 on small networks, and the constrained model with moderate depth AE2_l1 on large networks. For cannot-link constraints, the deep constrained model AE3_l1 is the top-ranked model on both small and large networks. Generally, a deeper network leads to a greater representational capacity (Goodfellow et al. 2016). Though it is difficult to know the reason for one architecture outperforming another with a high degree of certainty, the increased number of data points for must-link constraints in the large network compared to small network most likely requires the network to have an increased representational capacity. Thus, for must-link constraints, AE1_L1 is top-ranked on small networks, while on large networks a deeper version (AE2_L1) is the best performing.

**Table 5:** Average rank of autoencoder architectures over all small and large LFR networks, for must-link and cannot-link constraints

Architecture	Small networks		Large networks	
	Must-link constraints	Cannot-link constraints	Must-link constraints	Cannot-link constraints
AE1	3.0	4.5	2.5	4.0
AE1_L1	**1**.**0**	6.0	2.5	5.0
AE2	5.0	3.0	4.0	2.0
AE2_L1	2.0	4.5	**1**.**0**	6.0
AE3	6.0	2.0	6.0	3.0
AE3_L1	4.0	**1**.**0**	5.0	**1**.**0**

We turn now to the results for the traditional outlier detection methods, which are listed in Table 6. As can be seen for both small and large network, the SVM model achieves the highest scores on must-link constraints, while the IF model shows the best performance on cannot-link constraints. Generally, most models performed better in detecting noisy must-link constraints in small networks compared to large networks. However, the opposite trend is seen for cannot-link constraints, where we observe considerably higher scores on larger networks, except in the case of LOF model. Another trend that can be seen in Table 6 is significantly higher scores on networks with $O_n=10$% compared to networks with $O_n=50$% by most models, except for the IF and LOF models on must-link constraints in large networks. In summary, these results suggest that SVM and IF are the best performing models on must-link constraints and cannot-link constraints respectively across all networks. This can also be seen in Table 7, which reports the average ranking scores for the three alternative outlier detection models.

**Table 6:** Average AUC scores for Isolation Forest (IF), One-class SVM, and Local Outlier Factor (LOF) on LFR networks. We report overall scores, and scores on networks with differing overlap density $O_n$, for must-link and cannot-link sets separately

Outlier methods	Small networks						Large networks					
	Must-link constraints			Cannot-link constraints			Must-link constraints			Cannot-link constraints		
	Overall	$O_n=10$%	$O_n=50$%	Overall	$O_n=10$%	$O_n=50$%	Overall	$O_n=10$%	$O_n=50$%	Overall	$O_n=10$%	$O_n=50$%
IF	0.623	0.720	0.526	**0**.**868**	**0**.**915**	**0**.**820**	0.213	0.163	0.262	**0**.**932**	**0**.**968**	**0**.**896**
SVM	**0**.**691**	**0**.**751**	**0**.**631**	0.765	0.827	0.702	**0**.**554**	**0**.**572**	**0**.**536**	0.886	0.917	0.854
LOF	0.621	0.711	0.532	0.670	0.699	0.641	0.480	0.478	0.482	0.596	0.628	0.564

**Table 7:** Average ranks of Isolation Forest (IF), One-class SVM, and Local Outlier Factor (LOF) over all small and large LFR networks, for must-link and cannot-link constraints

Dataset	Must-link constraints			Cannot-link constraints		
Small networks	IF	SVM	LOF	IF	SVM	LOF
	2.3(2)	**1.3(1)**	2.4(3)	**1.0(1)**	2.0(2)	3.0(3)
Large networks	IF	SVM	LOF	IF	SVM	LOF
	3.0(3)	**1.1(1)**	1.9(2)	**1.0(1)**	2.0(2)	3.0(3)
Average Rank	2.5	**1**	2.5	**1**	2	3

#### Evaluating autoencoders for deep embeddings

**Methodology.** In this section we address the objective of finding the best autoencoder architectures for use as a deep embedding technique in combination with other outlier detection methods. The best candidates will be used later in Experiment 2 in section “Experiment 2: Evaluation of noise removal methods”. Specifically, we assess the performance of different autoencoder architectures with One-Class SVM and Isolation Forest (IF) models, which were the best performed conventional outlier detection models on must-link and cannot-link constraints respectively as described previously.

**Results.** Table 8 reports the average ranks achieved by different autoencoders (on the rows) in conjunction with the SVM and IF methods for detecting noise in must-link and cannot-link sets (on the columns). As can be seen from the results, unconstrained AE models outperform constrained ones as deep embedding technique in all cases. In particular, the deep unconstrained models AE3 shows the best scores, except for the case of SVMs on large networks, where the unconstrained model with moderate depth AE2 is the top-ranked model.

**Table 8:** Average ranks for autoencoder architectures when used as deep embeddings wit one-class SVM (on must-link constraints) and for Isolation Forest (on cannot-link constraints)

Architecture	Small networks		Large networks	
	Encoder + SVM	Encoder + IF	Encoder + SVM	Encoder + IF
AE1	3	2	2	3
AE1_L1	4	4	4	4
AE2	2	3	**1**	2
AE2_L1	5.5	5.5	5.5	5.5
AE3	**1**	**1**	3	**1**
AE3_L1	5.5	5.5	5.5	5.5

### Experiment 2: Evaluation of noise removal methods

**Methodology.** In the previous experiment, we focused on Phase 2 in Fig. 1 as a separate component. Now we evaluate the performance of the proposed architecture incorporating Phase 2. Given the best-performing outlier detection models and deep embedding functions identified in Experiment 1, we assess the performance of AC-SLPA community finding using each category of constraint cleaning process described in Section “Process for identifying noisy constraints” to identify the best option. Table 9 summarize the types of cleaning processes and models that are used in this experiment. Again we make use of 64 synthetic LFR networks.

**Table 9:** Different variations of the cleaning process using the best performing models from the Experiment 1, on must-link and cannot-link constraints respectively

Cleaning models	Must-link constraints	Cannot-link constraints
Small networks		
Hybrid (Autoencoder–Encoder Func.+IF)	AE1_L1	AE3+IF
Autoencoders(AE)	AE1_L1	AE3_L1
Encoder Func. + Outlier detection (SVM-IF)	AE3+SVM	AE3+IF
Outlier detection only (SVM-IF)	SVM	IF
Large networks		
Hybrid (Autoencoder–Encoder Func.+IF)	AE2_L1	AE3+IF
Autoencoders(AE)	AE2_L1	AE3_L1
Encoder Func. + Outlier detection (SVM-IF)	AE2+SVM	AE3+IF
Outlier detection only (SVM-IF)	SVM	IF

**Fig. 7:**
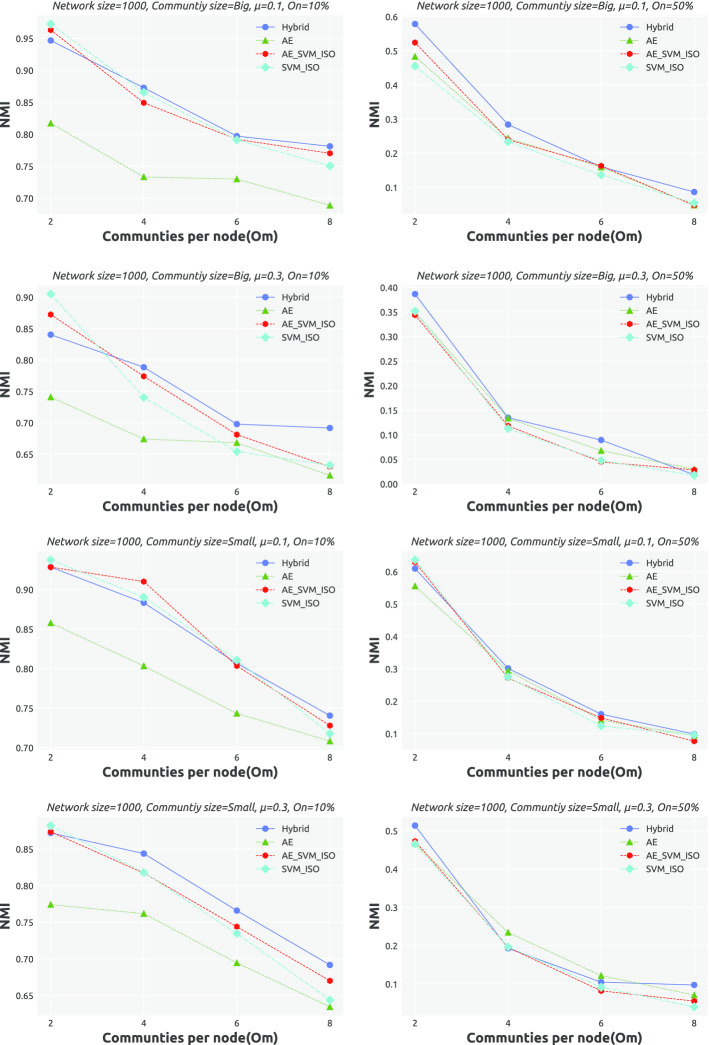
Performance of AC-SLPA using different constraint cleaning methods on *small synthetic networks*, containing both small and large communities, where the mixing parameter $\mu$ varies from 0.1 to 0.3. NMI values are plotted against the number of communities per node $(O_m)$

**Fig. 8:**
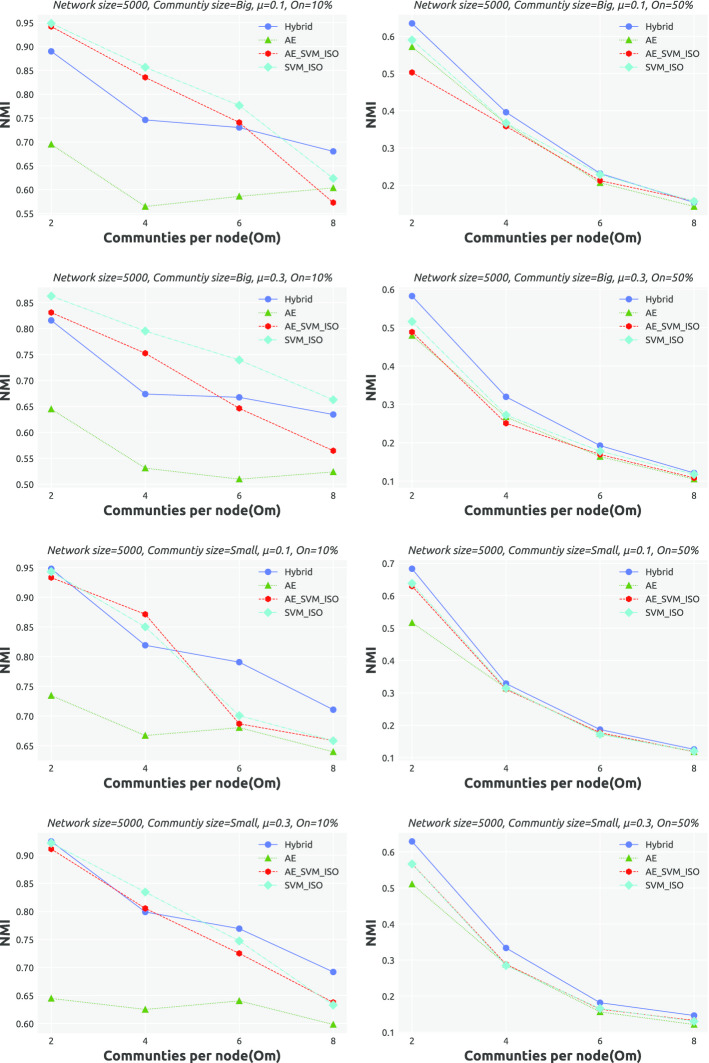
Performance of AC-SLPA using different constraint cleaning methods on *large synthetic networks*, containing both small and large communities, where the mixing parameter $\mu$ varies from 0.1 to 0.3. NMI values are plotted against the number of communities per node $(O_m)$

**Results.** Tables 10 and 11 provide an overview of how the performance of AC-SLPA with various cleaning methods changes on synthetic networks. Recall that these networks vary in terms of mixing parameter $\mu$, overlapping diversity $O_m$, overlapping density $O_n$, and the size of both the networks themselves and their ground truth communities. Each table entry includes the average NMI score of AC-SLPA combined with each cleaning methods (on the rows) over networks with specific parameters (on the columns). The best score is highlighted in bold. The detailed NMI scores are shown in Figs. 7 and 8, which indicate the agreement between the obtained communities in each case and the corresponding ground truth.

Generally, increasing the value of $\mu$ results in lower NMI scores for all algorithms, due to the increased proportion of inter-community edges that lead to weakly-defined community structure. As can be seen from Tables 10 and 11, compared to the case of $\mu =0.1$, the average NMI scores of all algorithms considerably decreased on small networks with $\mu =0.3$. In both cases of $\mu$, we can see that AC-SLPA with the Hybrid method outperformed other methods on small and large networks. As for examining the performance on networks with small and large communities, we can see that all algorithms show higher average NMI scores for small community networks compared to large community networks. In addition, we notice that AC-SLPA with the hybrid method shows the best performance on all networks, except for large networks with large communities.

Now we investigate the effect of two network properties, overlapping diversity $O_m$ and overlapping density $O_n$, on the performance of all algorithms. As we can see from Tables 10 and 11, the quality of obtained communities of all algorithms consistently decreases as the overlapping diversity and overlapping density increase. In most of the cases of $O_m$ and $O_n$, AC-SLPA with the Hybrid method outperform other methods except cases on $O_n=10\%$ and $O_m=4$ shows the second best scores after SVM_IF. Overall, in most cases of network parameters, the algorithms show higher average NMI scores on large networks compared to small networks, excluding AC-SLPA with AE method which shows a contrasting trend.

**Table 10:** Average NMI scores of AC-SLPA achieved using different cleaning processes on *small synthetic networks* with different network parameters

Cleaning process	Network parameters									
	$\mu$		Comm. Size		$O_n$		$O_m$			
	0.1	0.3	Small	Large	10%	50%	2	4	6	8
Hybrid	**0**.**565**	**0**.**483**	**0**.**538**	**0**.**51**	**0**.**810**	**0**.**239**	**0**.**710**	**0**.**538**	**0**.**448**	**0**.**401**
AE	0.506	0.440	0.497	0.449	0.728	0.218	0.631	0.485	0.416	0.361
AE_SVM_IF	0.553	0.463	0.525	0.49	0.800	0.215	0.701	0.522	0.432	0.376
SVM_IF	0.547	0.458	0.522	0.483	0.797	0.208	0.701	0.517	0.424	0.369

**Table 11:** Average NMI scores of AC-SLPA achieved using different cleaning processes on *large synthetic networks* with different network parameters

Cleaning Process	Network Parameters									
	$\mu$		Comm. Size		$O_n$		$O_m$			
	0.1	0.3	Small	Large	10%	50%	2	4	6	8
Hybrid	**0**.**566**	**0**.**530**	**0**.**567**	0.529	0.768	**0**.**328**	**0**.**763**	0.552	**0**.**469**	**0**.**408**
AE	0.474	0.426	0.464	0.435	0.618	0.281	0.6	0.453	0.39	0.357
AE_SVM_IF	0.544	0.503	0.539	0.508	0.757	0.290	0.726	0.559	0.44	0.369
SVM_IF	0.559	0.527	0.543	**0**.**543**	**0**.**785**	0.301	0.748	**0**.**572**	0.464	0.388

Table 12 summarizes the average ranks based on NMI scores for all algorithms on the synthetic networks. Each table entry shows the average rank of AC-SLPA with a cleaning method (on the columns) for different sizes of synthetic networks (on the rows). The average ranks based on NMI scores for each individual network is shown in Figs. 7 and 8. As we can see, AC-SLPA with the Hybrid method achieved the best rank on both small and large networks. The second-best algorithms with AE_SVM_IF method on small networks and with SVM_IF method on large networks. AE_SVM_IF and SVM_IF show approximately comparable performance on small networks, however the difference in performance between both methods grows higher on large networks.

**Table 12:** Average ranks of NMI scores for AC-SLPA achieved using different cleaning methods on small and large synthetic networks

Network category	Hybrid	AE	AE_SVM_IF	SVM_IF
Small networks	**1.6(1)**	3.2(4)	2.6(2)	2.7(3)
Large networks	**1.6(1)**	3.8(4)	2.7(3)	2.0(2)

**Fig. 9:**
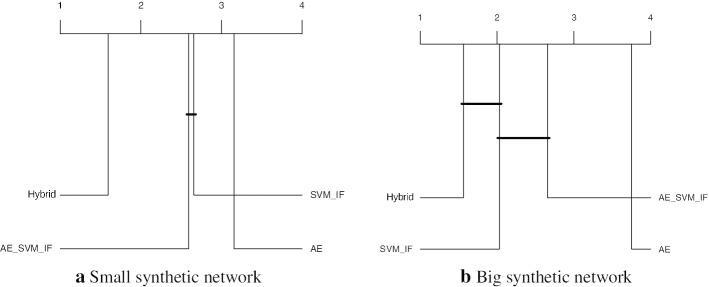
Critical difference plots from Friedman aligned rank test with Finner *p* value correction with significance level $\alpha = 0.05$ comparing Hybrid, AE_SVM_IF, SVM_IF and AE algorithms on the small and big synthetic networks. Algorithms which are not connected with the horizontal dark line are significantly different than each other. Lower rank indicates an overall better performance

To further understand the performance differences, we perform a Friedman aligned rank test with the Finner *p* value correction (García et al. 2010) to compare the above methods. The critical difference plots with a significance value $\alpha = 0.05$ of the test results are shown in Fig. 9, where the vertical lines indicate the corresponding algorithm’s rank. The algorithms which are not connected with the black horizontal line are significantly different with the mentioned significance level. In the case of the small synthetic networks, the Hybrid method was found to be significantly better than the other three methods. On the other hand, for big networks, this method was found to be significantly better than AE_SVM_IF and AE.

### Experiment 3: End-to-end evaluation

**Methodology.** In the previous section, we compared different cleaning methods as they were integrated into the overall architecture as can be seen in Fig. 1. The best performing cleaning process identified was the Hybrid method. We term this overall architecture AC-SLPA with Hybrid cleaning. In the following sections, we compare this architecture to the baseline algorithms, SLPA and AC-SLPA, without any constraint cleaning on both small and large synthetic networks.

**Fig. 10:**
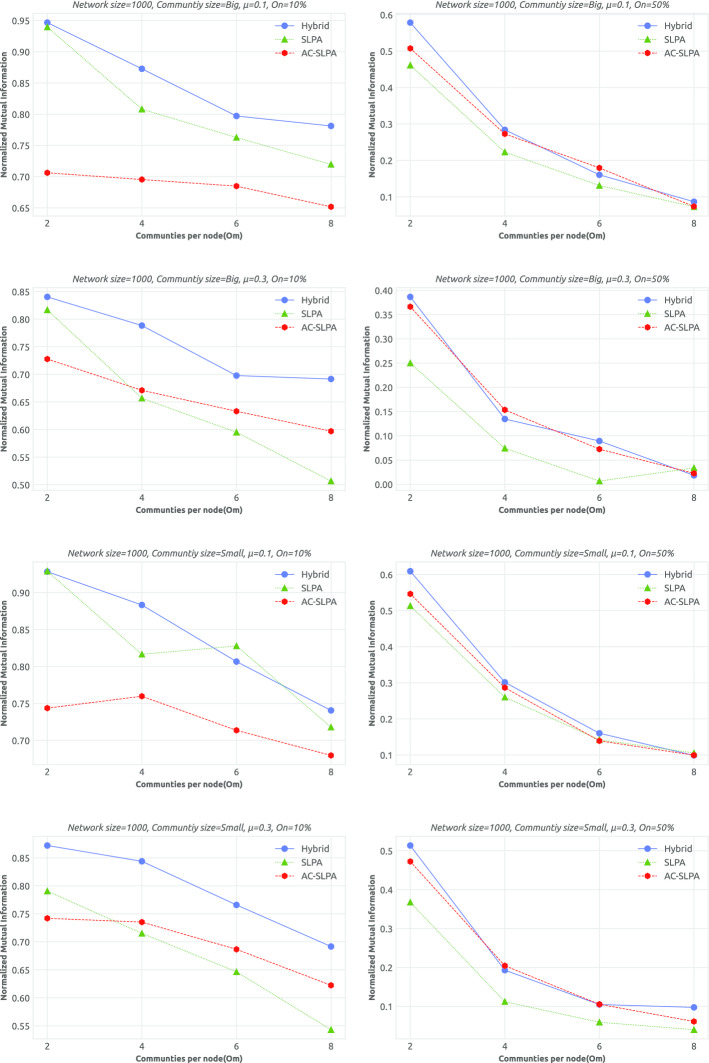
Performance of AC-SLPA using the hybrid cleaning process compared to SLPA and AC-SLPA with noisy pairwise constraints on *small synthetic networks*, containing both small and large communities, where the mixing parameter $\mu$ varies from 0.1 to 0.3. NMI values are plotted against the number of communities per node $(O_m)$

**Fig. 11:**
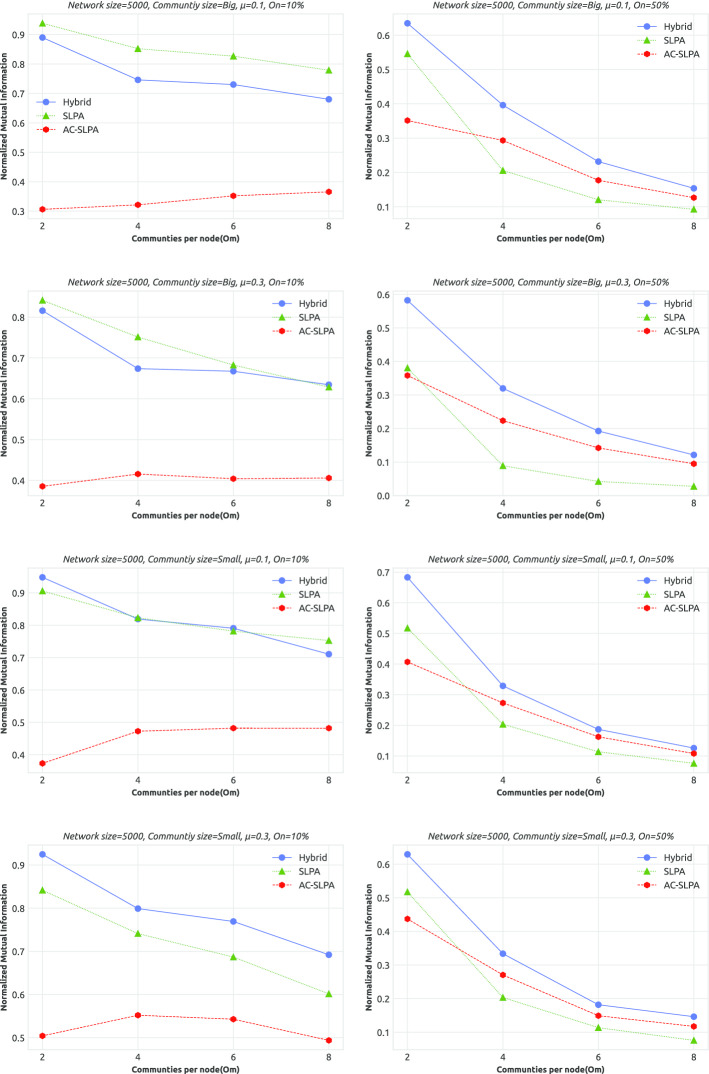
Performance of AC-SLPA using Hybrid cleaning method compared to SLPA and AC-SLPA with noisy pairwise constraints on *large synthetic networks*, containing both small and large communities, where the mixing parameter $\mu$ varies from 0.1 to 0.3. NMI values are plotted against the number of communities per node $(O_m)$

**Results.** We assess the quality of the obtained communities by AC-SLPA with hybrid (top-ranked cleaning process) compared to AC-SLPA and SLPA from the perspective of different network parameters as illustrated in Tables 13 and 14. The NMI scores of each network are reported in Figs. 10 and 11 on small and large networks respectively.

As can be seen from the Tables 13 and 14, AC-SLPA with Hybrid cleaning significantly outperformed other algorithms in most cases of networks parameters. For instance, in high mixing parameters large networks, AC-SLPA with the Hybrid method shows significantly higher score with NMI=0.530 compared to AC-SLPA and SLPA with NMI=0.343 and NMI=0.451 respectively. Similarly, AC-SLPA with the Hybrid method beats the other algorithms in most overlapping density ($O_n$) and overlapping diversity ($O_m$) cases, except on large networks with low overlapping density. SLPA shows slightly better average NMI score than AC-SLPA with cleaning process, with NMI=0.777 and NMI=0.768 respectively. In addition, we notice that the AC-SLPA with hybrid cleaning and SLPA show higher average NMI scores on large networks compared to small networks.

**Table 13:** Average NMI scores of AC-SLPA using Hybrid cleaning method compared to SLPA and AC-SLPA with noisy pairwise constraints on *small synthetic networks* with different parameters

Algorithm	Network parameters									
	$\mu$		Comm. Size		$O_n$		$O_m$			
	0.1	0.3	Small	Large	10%	50%	2	4	6	8
Hybrid	**0**.**565**	**0**.**483**	**0**.**538**	**0**.**510**	**0**.**809**	**0**.**239**	**0**.**709**	**0**.**538**	***0***.***448***	**0**.**401**
AC-SLPA	0.484	0.43	0.475	0.438	0.691	0.223	0.602	0.472	0.402	0.351
SLPA	0.527	0.388	0.474	0.441	0.737	0.178	0.634	0.458	0.396	0.342

**Table 14:** Average NMI scores of AC-SLPA using the hybrid cleaning process, SLPA and AC-SLPA with noisy pairwise constraints on *large synthetic networks* with different parameters

Algorithm	Network parameters									
	$\mu$		Comm. Size		$O_n$		$O_m$			
	0.1	0.3	Small	Large	10%	50%	2	4	6	8
Hybrid	**0**.**566**	**0**.**530**	**0**.**567**	**0**.**529**	0.768	**0**.**328**	**0**.**763**	**0**.**552**	**0**.**469**	**0**.**408**
AC-SLPA	0.316	0.343	0.364	0.295	0.429	0.231	0.390	0.353	0.301	0.357
SLPA	0.533	0.451	0.497	0.488	**0**.**777**	0.208	0.686	0.484	0.421	0.369

On the small networks, the performance of AC-SLPA without any cleaning process shows slightly better than SLPA in most cases. In contrast, the performance of AC-SLPA is significantly affected by noisy pairwise constraints on the large networks, where the average NMI score is consistently lower compared to SLPA. Overall, the best NMI scores across all algorithms are shown on networks with low overlapping density, as we might expect. For instance, we can see from Figs. 10 and 11 that the Hybrid method achieves higher NMI scores on most networks with low overlapping density compared to other algorithms, and the scores drop in high overlapping density case, in particular on small networks. On the large networks, the performance of the Hybrid method is considerably higher and more stable as the overlapping diversity increases, when compared to AC-SLPA and SLPA. Table 15 lists the average ranks of NMI scores of all algorithms on small and large networks, which shows the average ranks (lower values are better) of an algorithm (on the columns) over different size of synthetic networks (on the rows). The best scores are shown in boldface. As we see in Table 15, AC-SLPA with Hybrid cleaning method achieved the best rank score on both small and large networks, followed by SLPA on small networks and AC-SLPA on large networks.

**Table 15:** Average ranks of NMI scores of AC-SLPA using the hybrid cleaning process, SLPA and AC-SLPA with noisy pairwise constraints on small and large synthetic networks

Network category	SLPA	AC-SLPA	Hybrid
Small networks	2.2(2)	2.5(3)	**1.3(1)**
Large networks	2.6(3)	2.1(2)	**1.3(1)**

**Fig. 12:**
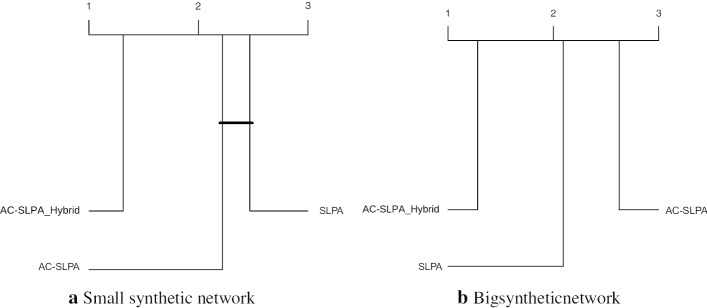
Critical difference plots from Friedman aligned rank test with Finner *p* value correction with significance level $\alpha = 0.05$ comparing Hybrid (the best performing variant from the previous experiment), SLPA and AL-SLPA algorithms on small and large synthetic networks. Algorithms which are not connected with the horizontal dark line are significantly different than each other. Lower ranks indicate better overall performance

As in Section “Experiment 2: Evaluation of noise removal methods”, we perform a Friedman aligned rank test with the Finner *p* value correction to support a multiple comparison test between the three methods above. The critical difference plots of the results with a significance level of $\alpha = 0.05$ are shown in Fig. 12. In the case of both the small and large networks, the AC-SLPA with Hybrid method performed significantly better than the other two methods.

### Experiment 4: Real-world networks

**Methodology.** We now discuss our final experiment on three real-world networks (Amazon, YouTube, DBLP). We use the same setup employed in Sections “Experiment 2: Evaluation of noise removal methods” and “Experiment 3: End-to-end evaluation” to examine the performance of each cleaning method after integration with AC-SLPA. Note that we employ the same models used with large synthetic networks, see Table 9. As baselines we consider AC-SLPA without cleaning (i.e. keeping noisy constraints), and the purely unsupervised algorithm SLPA. We also compare AC-SLPA with the best cleaning method to other baseline algorithms which are OSLOM (Lancichinetti et al. 2011), MOSES (McDaid and Hurley 2010), COPPRA (Adamcsek et al. 2006) on real-world networks in Table 20.

**Results.** Table 19 lists the NMI scores for each algorithm (columns) on each network (rows). The last row reports the average rank score of each algorithm. When comparing the performance of AC-SLPA with different cleaning methods to the baseline algorithms, we can see AC-SLPA with the Hybrid method achieves the best NMI scores on YouTube and DBLP networks (with NMI=0.818 and NMI=0.921 respectively). On the YouTube network, the performance of the AC-SLPA with Hybrid cleaning method increases significantly with a small amount of supervision. The next best performer is the AC-SLPA with the AE method, followed by SLPA and AC-SLPA with noisy pairwise constraints. All algorithms achieve their highest NMI scores on the YouTube dataset.

**Table 19:** Average NMI scores of AC-SLPA using different cleaning methods (Hybrid, AE, AE_SVM_IF, and SVM_IF), SLPA and AC-SLPA with noisy pairwise constraints on three real-world networks. Average ranks across the networks are also reported

Network	SLPA	AC-SLPA	AC-SLPA_Hybrid	AC-SLPA_AE	AC-SLPA_AE_SVM_IF	AC-SLPA_SVM_IF
Amazon	**0**.**957**	0.956	0.956	0.952	0.951	0.955
YouTube	0.627	0.778	**0**.**818**	0.778	0.751	0.751
DBLP	0.897	0.892	**0**.**921**	0.906	0.889	0.893
Avg. Ranks	3.3	3.7	**1**.**3**	3.0	5.3	4.3

However, the cleaning methods fail to lead to any improvement over the baselines in the case of the Amazon network. After investigating these results in more detail, we notice two behaviors which frequently occur. Firstly, far more must-link constraints than cannot-link constraints are selected by AC-SLPA. For example, the number of must-link constraints often exceed 2,000 pairs, while the selected cannot-link constraint set can contain fewer than 100 pairs. Secondly, all of the noisy constraints are in the cannot-link set, and the number of incorrectly-labelled pairs exceeds the number of correctly-labelled pairs. This situation renders noisy detection almost impossible using most outlier detection methods.

Now we compare the performance of AC-SLPA with the Hybrid method to an additional set of baseline algorithms: OSLOM (Lancichinetti et al. 2011), MOSES (McDaid and Hurley 2010), and COPRA (Adamcsek et al. 2006). From the results shown in Table 20, we see that OSLOM achieves the highest NMI score on the Amazon network. However, AC-SLPA with Hybrid cleaning achieves the highest NMI score on the YouTube and DBLP networks. Table 20 also reports the average ranks for NMI scores across all algorithms on the real-world networks. This shows the average rank (lower values are better) of an algorithm (columns) over networks (rows). As can be seen, AC-SLPA with Hybrid cleaning achieved the best overall rank score, with SLPA and OSLOM next best.

**Table 20:** Average NMI scores of AC-SLPA using Hybrid cleaning methods compared to other baseline methods (SLPA, OSLOM, MOSES, COPRA) on three real-world networks. Average ranks across the networks are also reported

Network	AC-SLPA_Hybrid	SLPA	OSLOM	MOSES	COPRA
Amazon	0.956	0.957	**0**.**967**	0.908	0.962
YouTube	**0**.**818**	0.627	0.449	0.421	0.191
DBLP	**0**.**921**	0.897	0.849	0.771	0.914
Avg. Ranks	**2.0(1)**	2.7(2.5)	2.7(2.5)	4.7(5)	3.0(4)

**Fig. 13:**
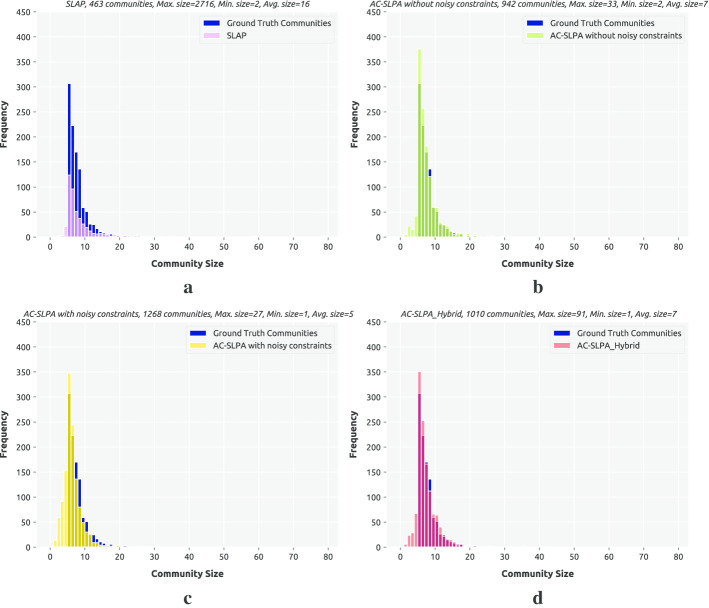
Community size distribution for communities produced by SLPA, AC-SLPA with noisy pairwise constraints, and AC-SLPA with Hybrid cleaning on the YouTube network, compared to the ground truth community size distribution

**Fig. 14:**
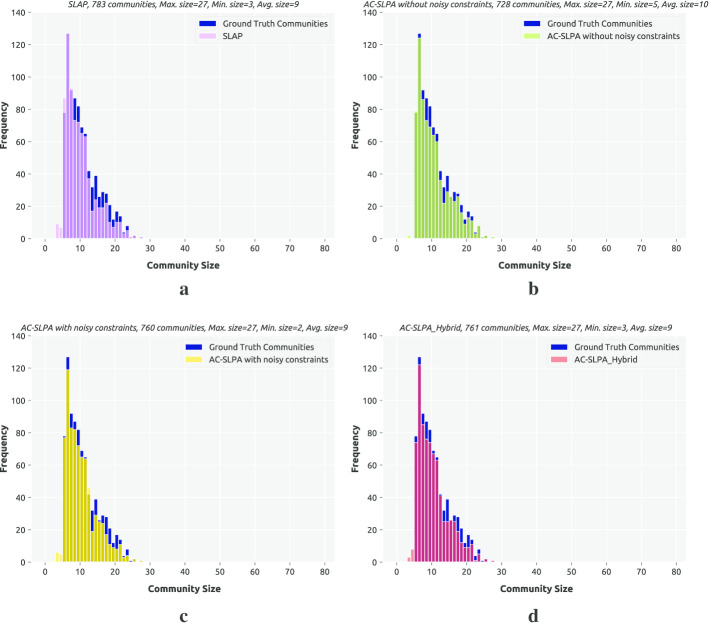
Community size distribution for communities produced by SLPA, AC-SLPA with noisy pairwise constraints, and AC-SLPA with Hybrid cleaning on the Amazon network, compared to the ground truth community size distribution

**Fig. 15:**
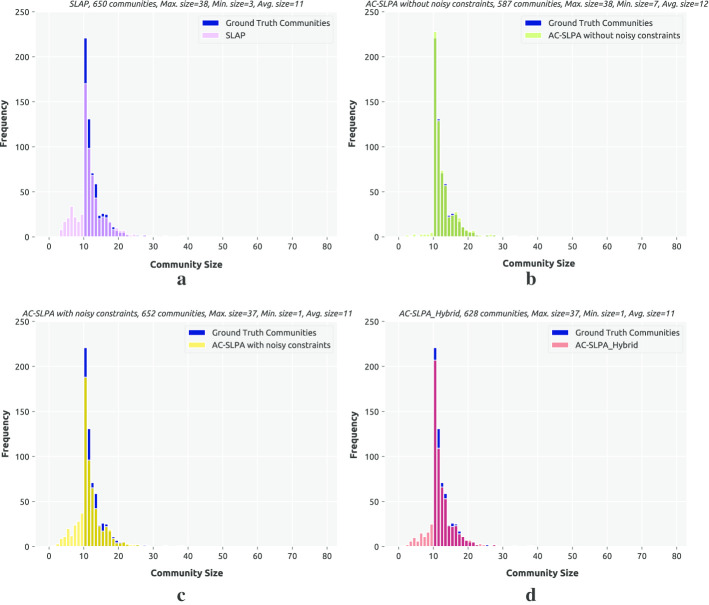
Community size distribution for communities produced by SLPA, AC-SLPA with noisy pairwise constraints, and AC-SLPA with Hybrid cleaning on the DBLP network, compared to the ground truth community size distribution

**Topological evaluation.** Finally, we explore the obtained communities’ topological properties for the methods AC-SLPA with Hybrid cleaning, AC-SLPA with and without noisy constraints, and SLPA. Specifically, we look at the community size distributions, as shown in Figs. 13, 14, and 15. We compare the size distribution of the communities produced by each algorithm against the distribution for the ground truth communities for each network (i.e., the reference distribution). Since all of these algorithms include a random component and were run 10 times, we focus on the run with the highest NMI score in each case. To compare distributions, we use a two-sample Kolmogorov–Smirnov test (KS) (Massey 1951). This is a non-parametric statistical test to compare two cumulative distributions, which calculates the maximum difference between them. We can then compute a *p* value based on this maximum distance and the sample sizes. The null hypothesis is that both distributions are identical. This hypothesis is rejected when the *p* value is small (< 0.05), and the distance value is high.

Table 21 reports the KS results for all the algorithms on the real-world networks. We observe that the *p* values for all variants of AC-SLPA on the Amazon network indicate that their size distributions are the same as the reference distribution, unlike SLPA. In term of the distance values, we can see that the distribution for AC-SLPA without noisy constraints is closest to the reference distribution. On the YouTube network we observe that, according to the *p* values, all algorithms’ distributions are not the same as the reference. However, when we inspect the distance values, again AC-SLPA without noisy constraints has the lowest score. We can also see that using Hybrid method with AC-SLPA reduces the distance value significantly. The same observation also applies for the DBLP network, although the *p* value for AC-SLPA without noisy constraints on this network is above 0.05.

Overall, we see that the semi-supervised approaches can successfully identify heterogeneously-sized communities present in the real-world networks as illustrated in Figs. 13, 14, and 15. Also, we notice that using the Hybrid method with AC-SLPA reduces the difference between the obtained communities and the ground-truth communities in terms of their size distributions, which results from the presence of noisy constraints.

**Table 21:** Kolmogorov-Smirnov distance between the community size distribution of the obtained results of SLPA, ACSLPA with and without noisy constraints, and ACSLPA_Hybrid on real-world networks against the community size distribution of the ground truth communities

Network	SLPA		ACSLPA with noise		ACSLPA_Hybrid		ACSLPA without noise	
	Distance	*p* value	Distance	*p* value	Distance	*p* value	Distance	*p* value
Amazon	0.076	0.016	0.059	0.113	0.050	0.252	0.033	0.772
YouTube	0.086	0.017	0.250	0.000	0.099	0.000	0.073	0.005
DBLP	0.222	0.000	0.222	0.000	0.141	0.000	0.032	0.899

## Conclusion

In this study, we have addressed the problem of handling noisy constraints in overlapping semi-supervised community detection, by treating them as outliers and use outlier detection models to find and remove them. Our primary contributions are four-fold: (1) a general architecture for semi-supervised community finding with noisy constraint filtering; (2) multiple designs of cleaning methodologies; (3) an investigation of outlier detection models for filtering, including deep learning models; (4) a comprehensive evaluation for each proposed cleaning methodology integrated in the context of community detection. Based on the experimental results, we found that the most effective approach was to employ a hybrid design of conventional and deep learning-based outlier detection models, in conjunction with the AC-SLPA algorithm. Using this approach makes the application of semi-supervised community finding approaches to real-world network scenarios more feasible as real annotations are always likely to be noisy which leads to poor performance when approaches that assume they will be clean are used. As future work, we will aim to explore the use of multiple noisy oracles (e.g a committee of human annotators), and how to resolve the disagreements which might arise between them.

## Data Availability

The datasets generated and analyzed during the current study are available online: https://github.com/elhamalghamdiUCD/Semi-Supervised-SLPA.

## Abbreviations

AC-SLPA: Active semi-supervised SLPA
NMI: Normalized mutual information
SLPA: Speaker-listener label propagation algorithm
*PC*−: Noisy pairwise constraints
*PC*+: Clean pairwise constraints
*ML*+: Clean must-link constraints
*ML*−: Noisy must-link constraints
*CL*+: Clean cannot-link constraints
*CL*−: Noisy cannot-link constraints
IF: Isolation forest
SVM: One class support vector machine
LOF: Local outlier factor
AE: Autoencoders
